# Supplementary material for: Alpha-synuclein stepwise aggregation reveals features of an early onset mutation in Parkinson’s disease
Source: Commun Biol. 2019 Oct 11;2:374. doi: 10.1038/s42003-019-0598-9 (PMC6789109; doi:10.1038/s42003-019-0598-9)
Supplement: Supplementary file 1 — Supplementary figures [file 42003_2019_598_MOESM1_ESM.pdf]

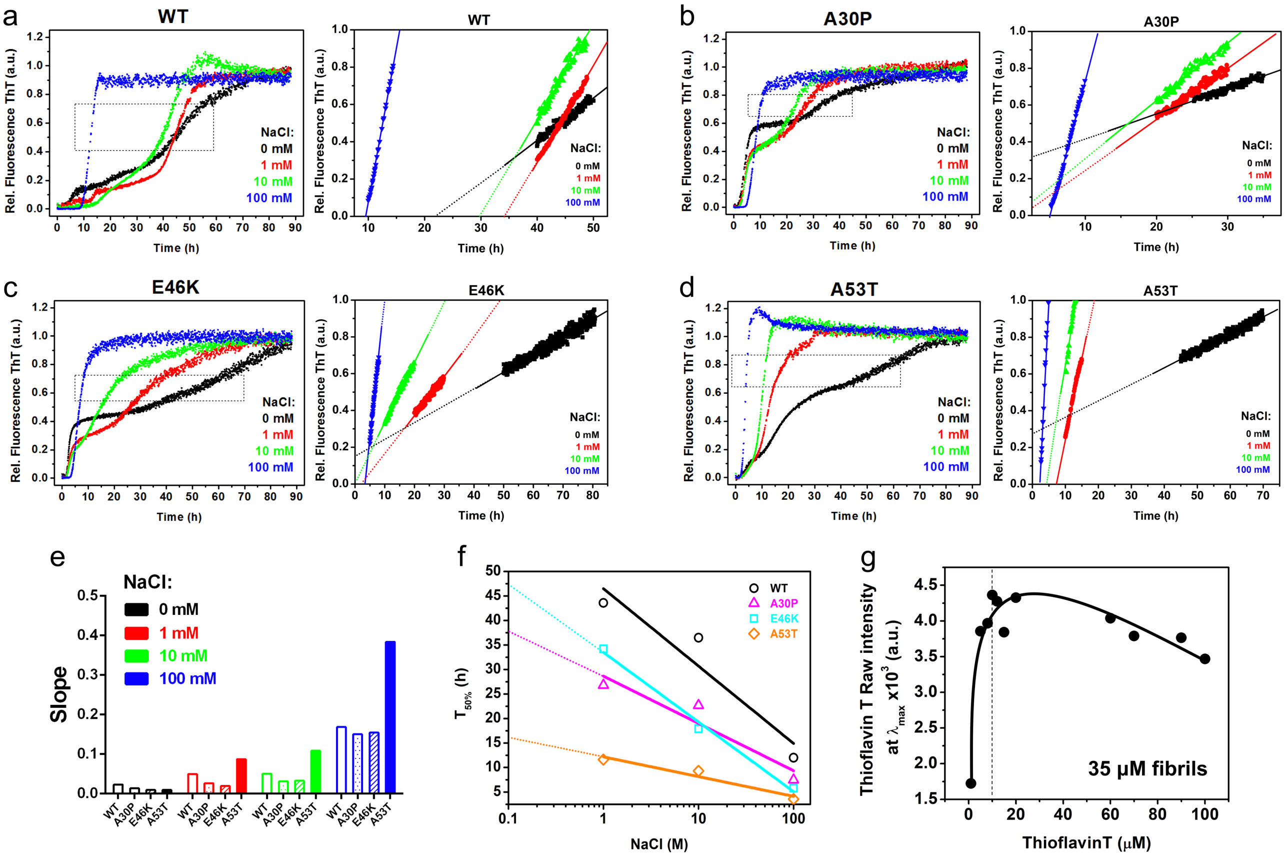

Supplementary Figure 1. (a-d) Linear regression of the points located in the exponential growth phase of the ThT kinetics (dashed rectangles) at different salt concentrations for the studied constructs. (e) Slope values extracted from linear regression for each variant at different concentrations of salt. (f) Half-time ($T_{50\%}$) in hours as a function of the studied sodium chloride concentrations for variants of αS (g) Thioflavin T (ThT) calibration to extract maximum concentration in which dye has a linear behavior (~ 8 μM).

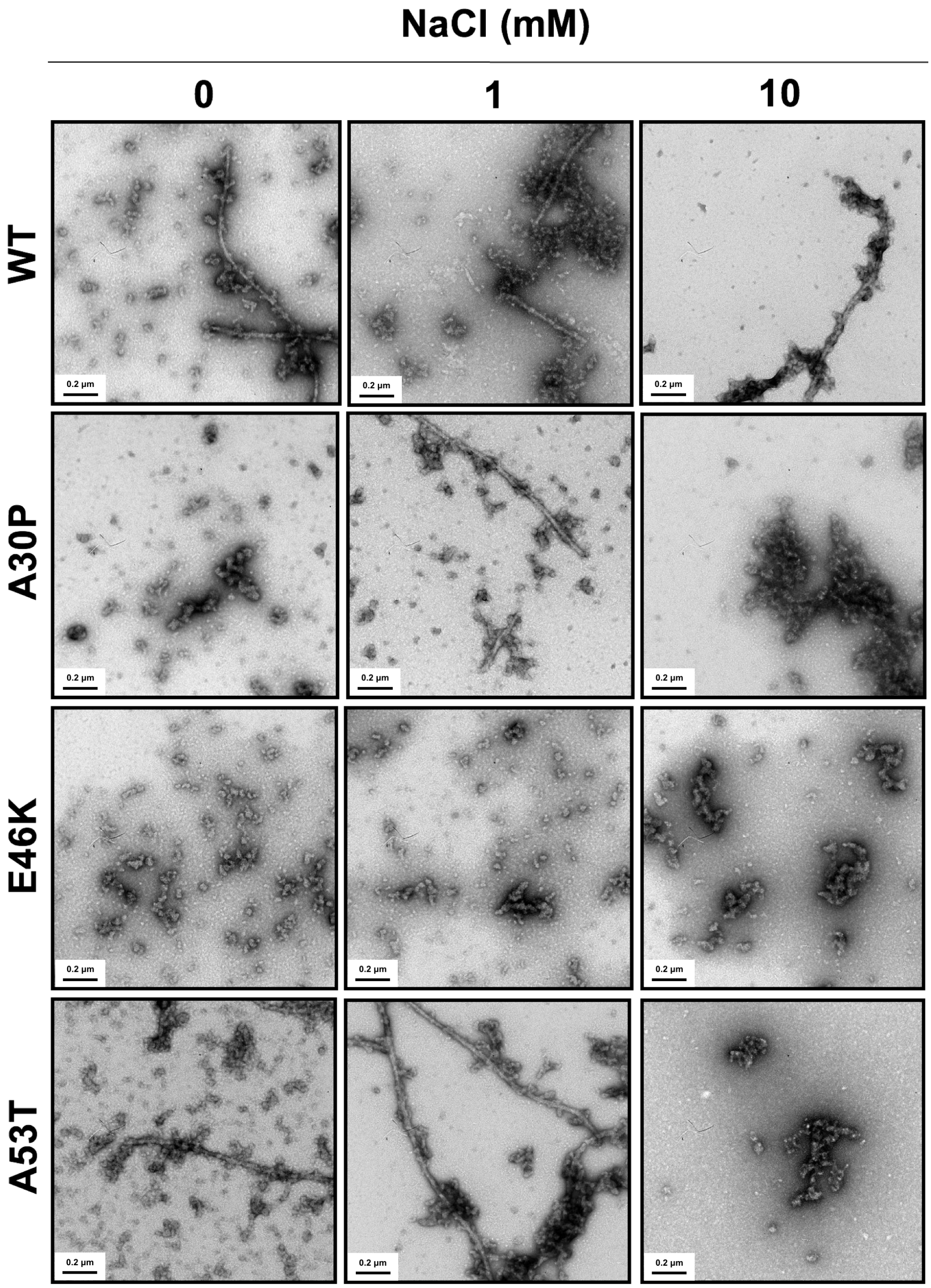

Supplementary Figure 2. Negatively stained electron micrographs of the soluble fractions obtained after ThT kinetic measurements of αS variants at different salt concentrations.

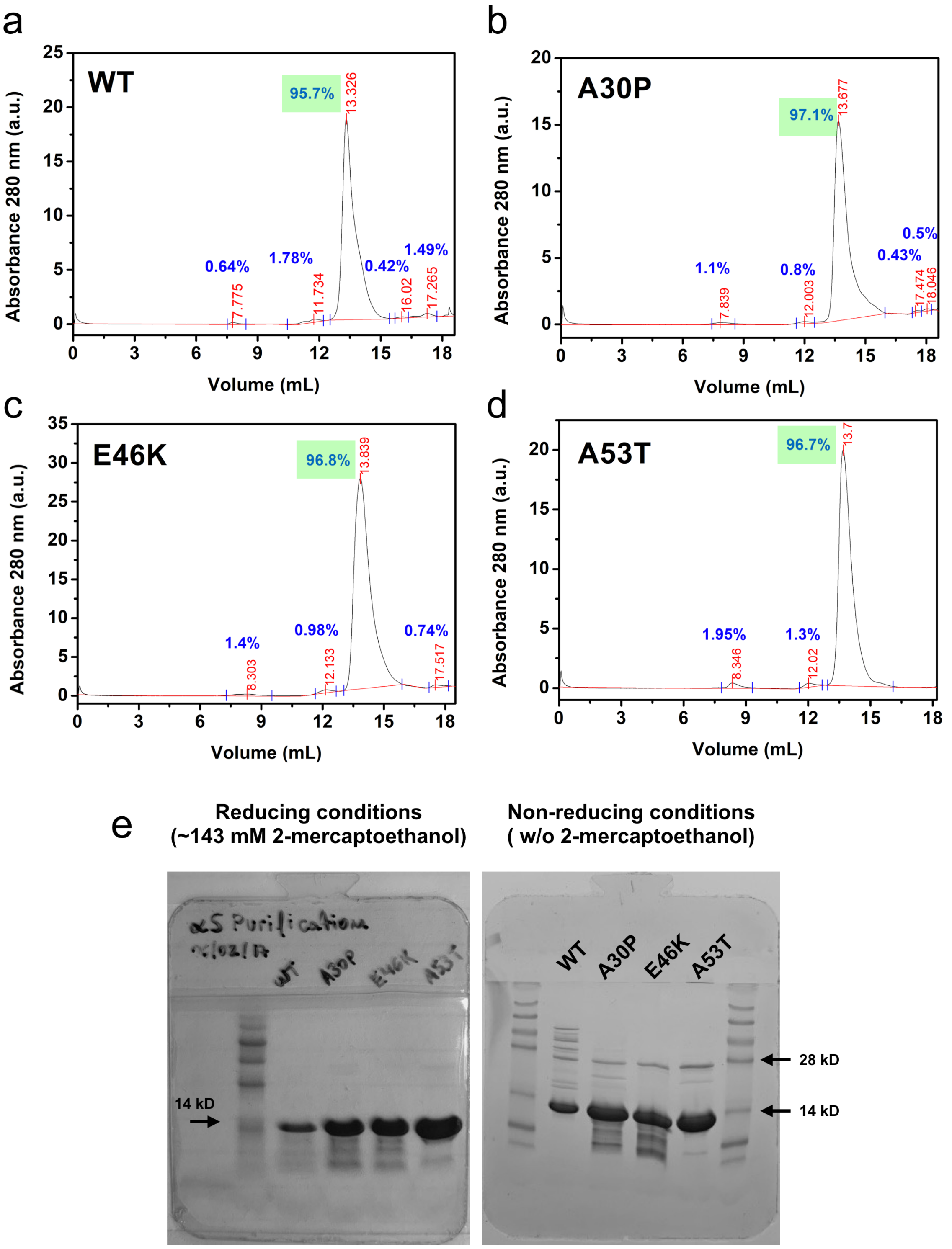

Supplementary Figure 3. Quality checks of protein preparations. (a-d) Peak integration analysis of SEC runs (Superdex 200) immediately after protein preparation. Red and blue values stand for retention volumes and percentage of corresponding peaks, respectively. (e) SDS-PAGE gels of purified proteins under reducing and non-reducing conditions.

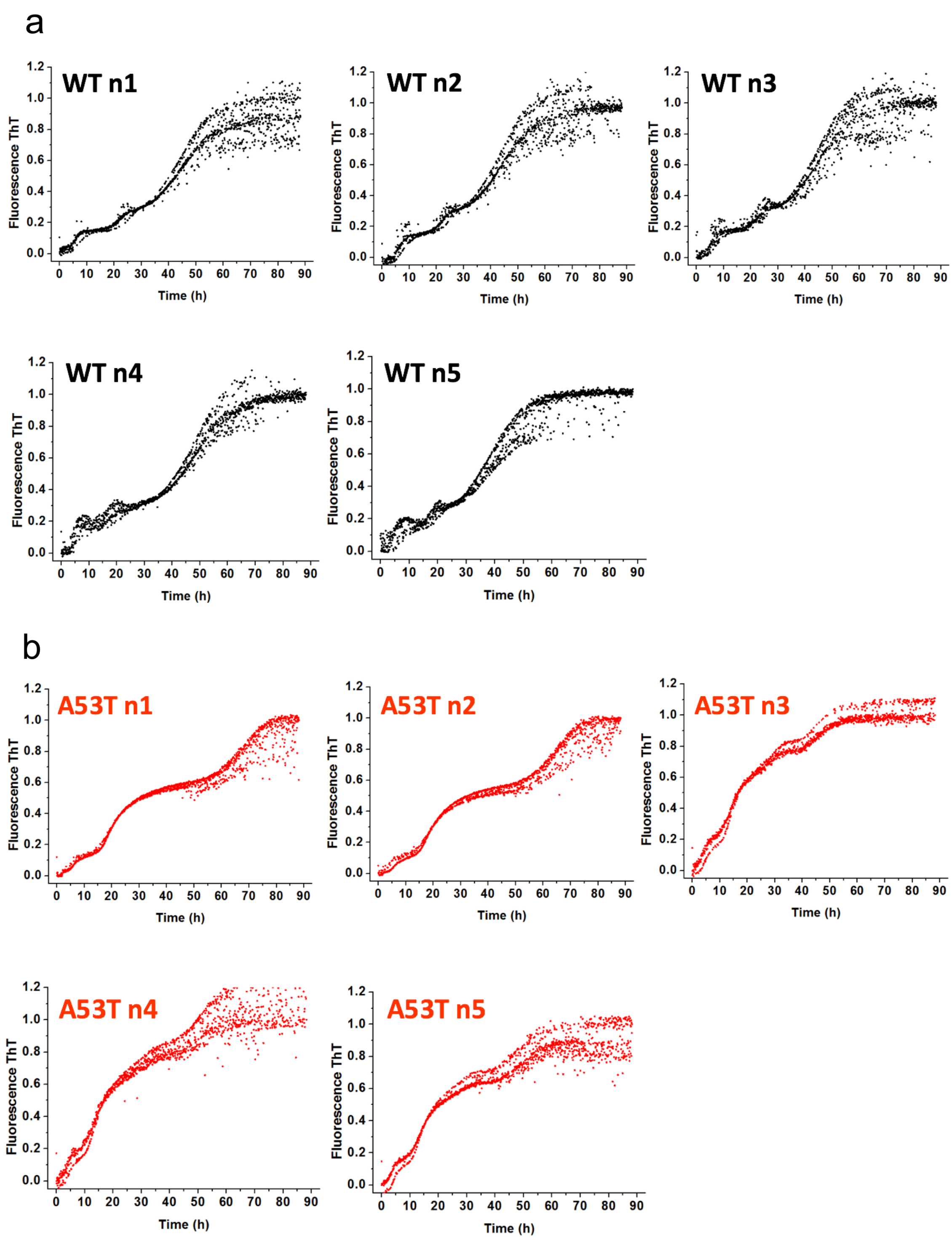

Supplementary Figure 4. Replicates (n1-n5) of two independent plates showing the self-consistency of the raw ThT kinetics and the multistep transition obtained for (a) wt and (b) A53T αS under the condition of no salt and agitation.

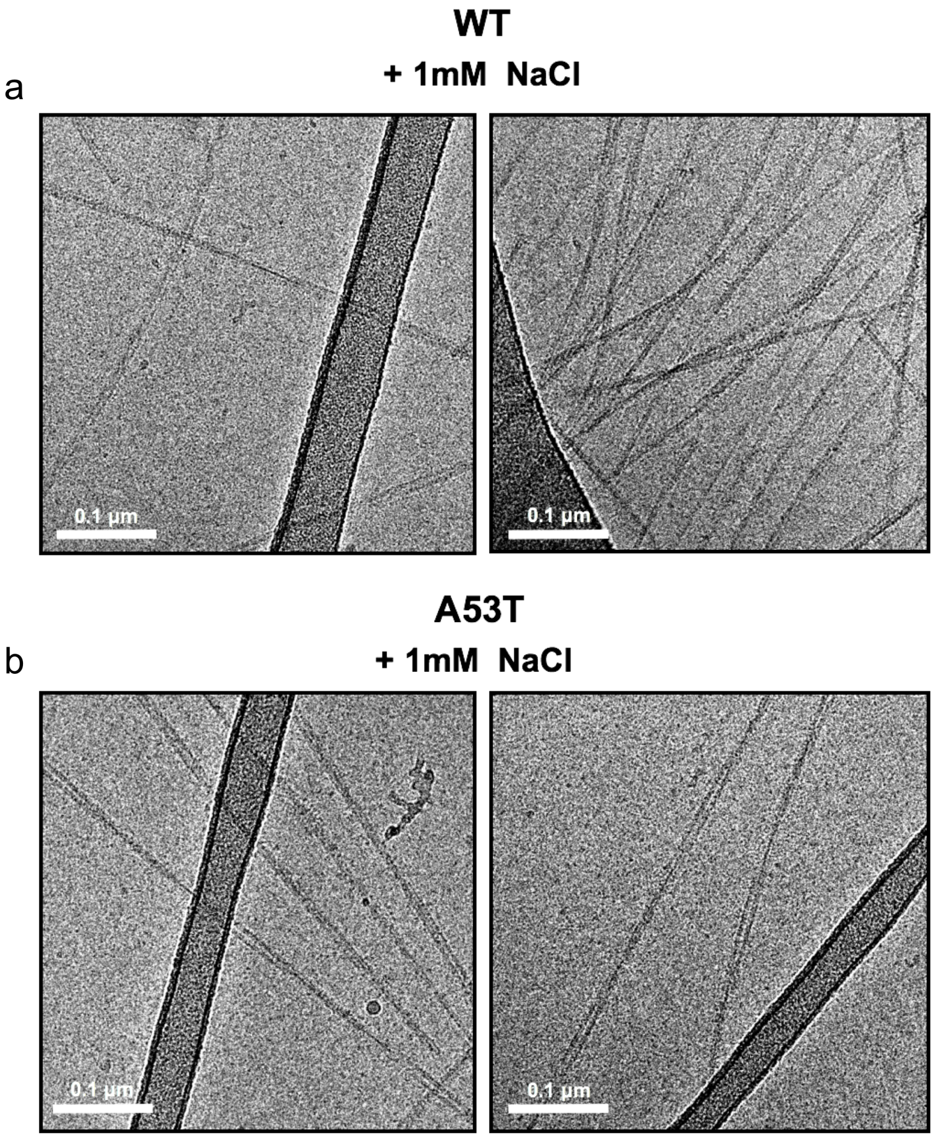

Supplementary Figure 5. Frozen hydrated micrographs of (a) wt and (b) A53T αS fibrils grown under the influence of 1 mM sodium chloride.

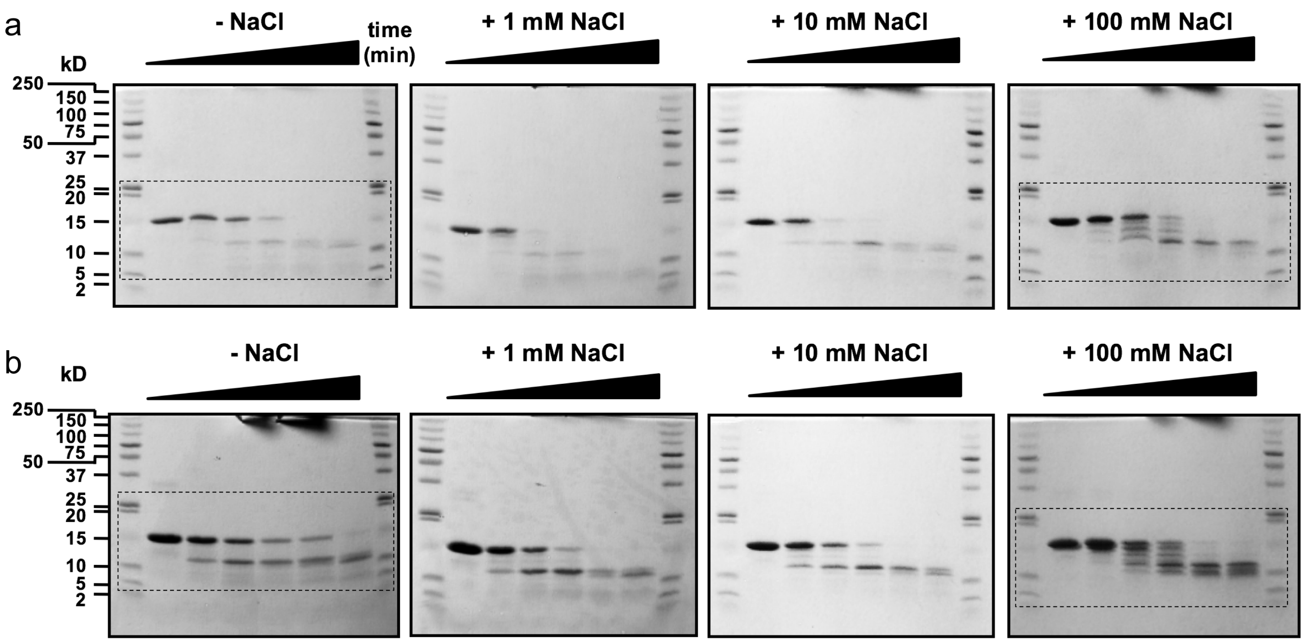

Supplementary Figure 6. Full SDS-PAGE gels (20%) showing the proteinase K degradation profiles of (a) wt and (b) A53T αS fibrils grown under the influence of different concentrations of sodium chloride. Lane 1: molecular standard; Lane 2: time 0; Lane 3: 1 min of digestion; Lane 4: 5 min of digestion; Lane 5: 10 min of digestion; Lane 6: 30 min of digestion; Lane 7: 60 min of digestion; Lane 8: molecular standard. The dashed lines show the extracted strips used in Fig. 5a, c.

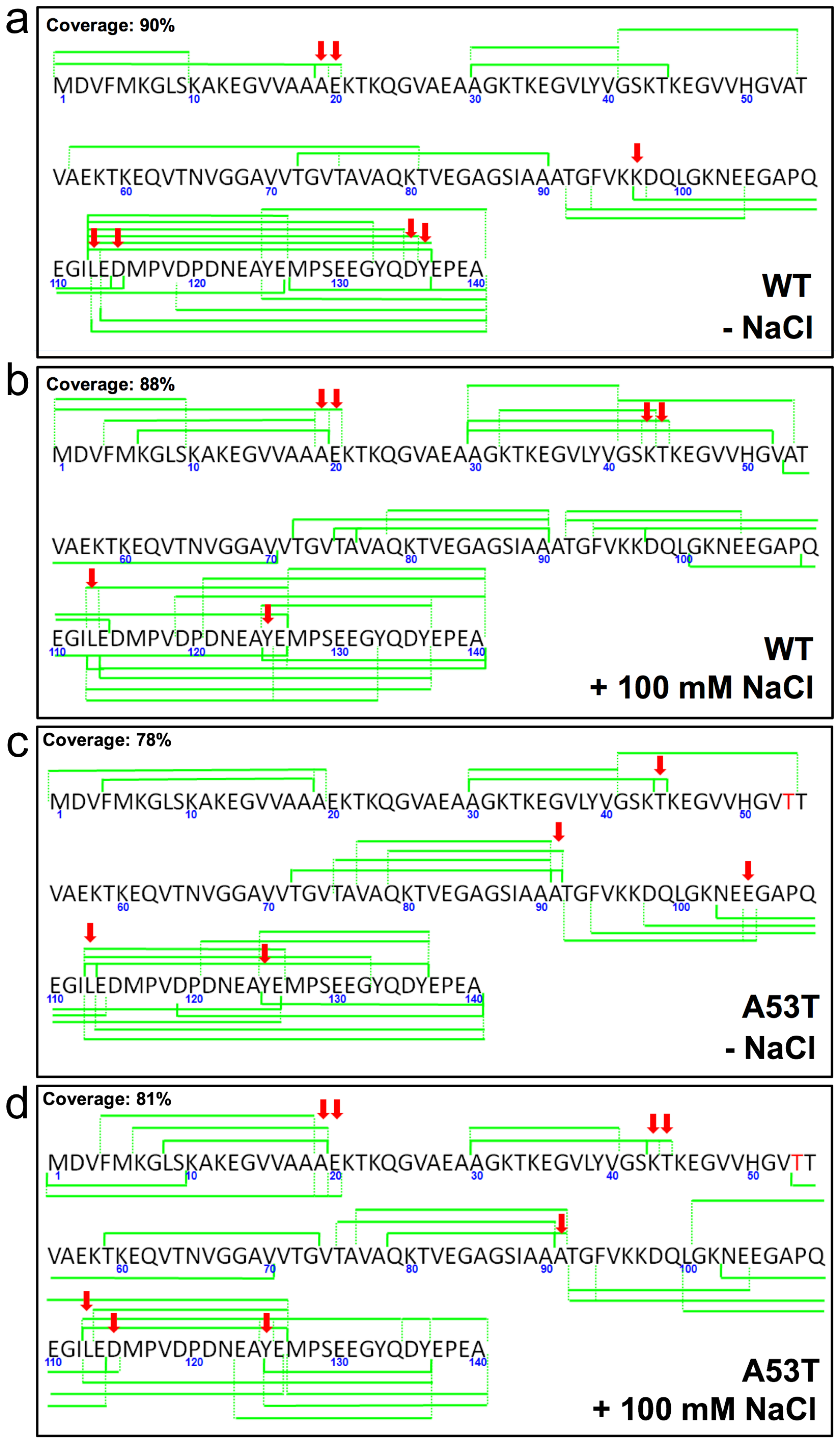

Supplementary Figure 7. Peptide coverage obtained on MS runs of (a, b) wt and (c, d) A53T in the absence or presence of 100 mM sodium chloride, respectively. Green lines show the identified peptides in the αS primary sequence. Red arrows represent prominent cuts. The Ala to Thr substitution at position 53 is red.

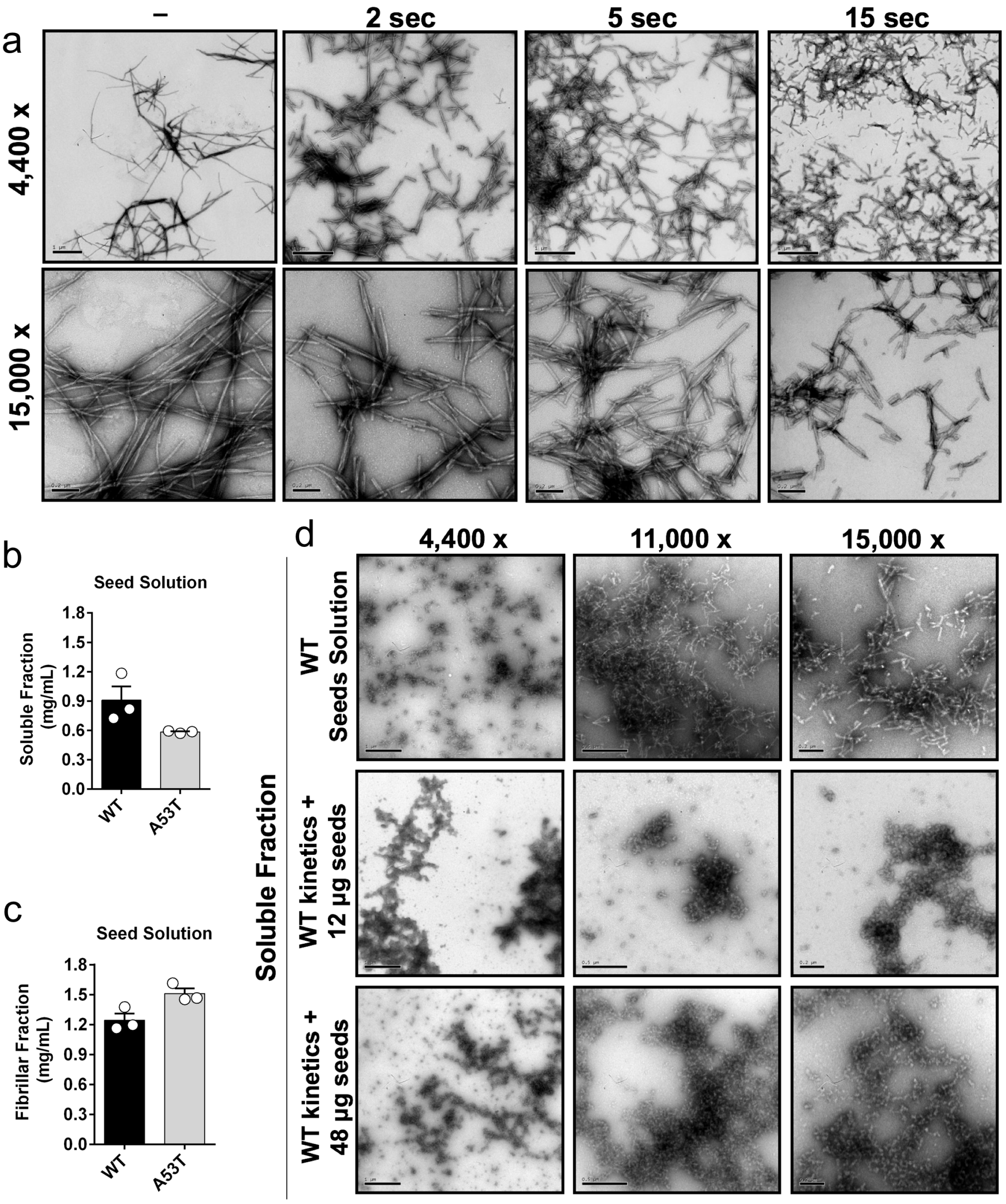

Supplementary Figure 8. (a) Before seed preparation, wt αS fibrils were exposed to 2, 5, and 15 s of ultrasound and imaged by negative staining electron microscopy to evaluate the formation of seeds. We chose the time of 15 s to prepare seeds for ThT kinetic runs. (b) Soluble and (c) fibrillar fractions of three independent wt and A53T seed preparations. (d) Negative staining electron micrographs of soluble fractions obtained from a solution of wt seeds and after wt ThT kinetic measurements with 12 or 48 µg of seeds (See Fig. 7f). Different magnification levels are shown to better visualize the formed species.

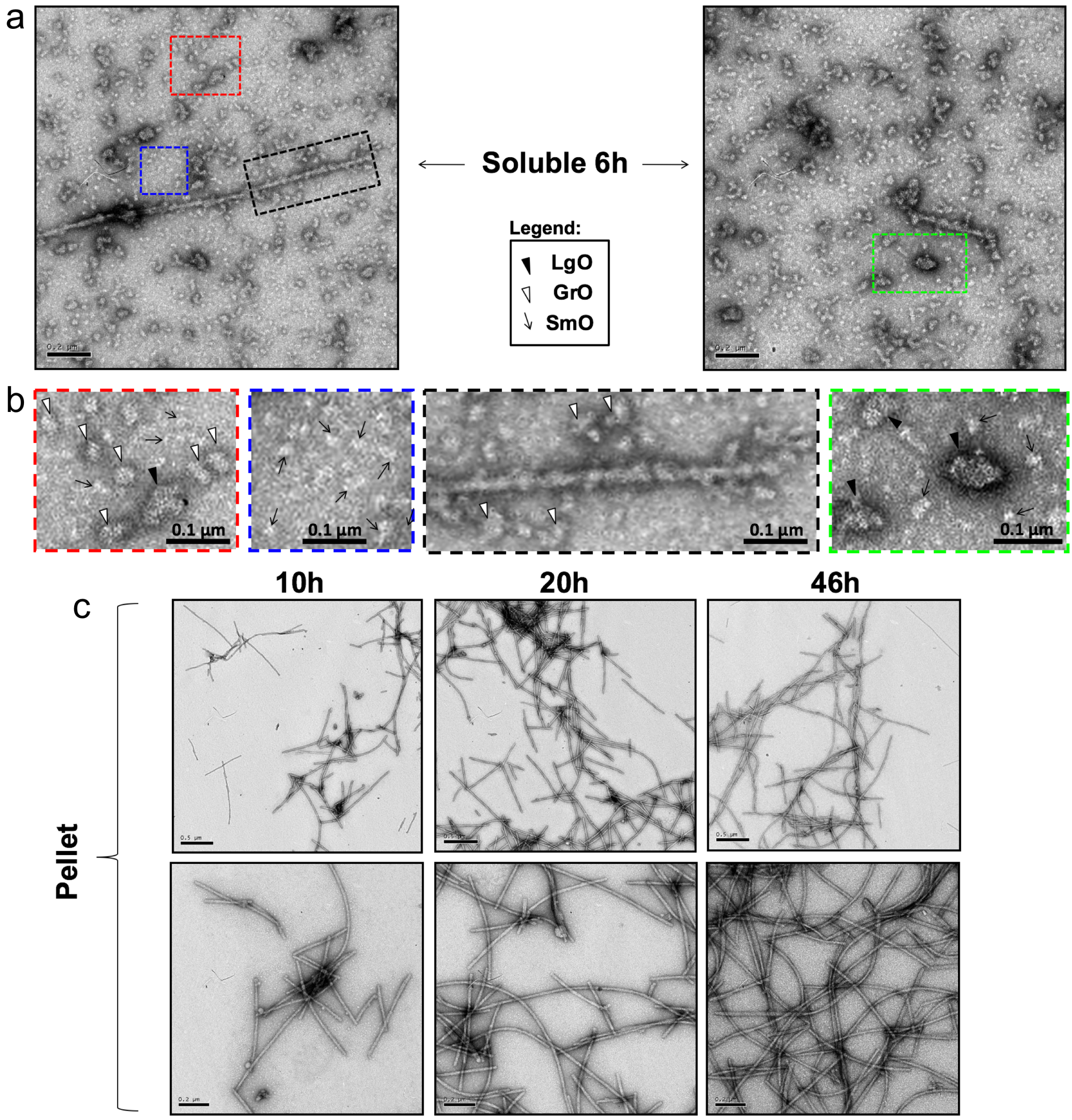

Supplementary Figure 9. (a) Negative staining electron micrographs of soluble species after 6 h of wt ThT kinetics. (b) Zoomed-in regions highlighted in (a). SmO - small oligomers; GrO - growing oligomers; LgO - large oligomers. (c) Pellet species obtained after 10, 20, and 46 h of wt ThT kinetics. Magnification: 6,500× (top), 15,000× (bottom).
