# Supplementary material for: Alpha-synuclein stepwise aggregation reveals features of an early onset mutation in Parkinson’s disease
Source: Commun Biol. 2019 Oct 11;2:374. doi: 10.1038/s42003-019-0598-9 (PMC6789109; doi:10.1038/s42003-019-0598-9)
Supplement: Supplementary file 2 — Description of Additional Supplementary Files [file 42003_2019_598_MOESM2_ESM.docx]

**Description of Additional Supplementary Files**

**File Name**: **Supplementary Data 1**

**Description**:   Source data file
