# Supplementary material for: Alpha-synuclein stepwise aggregation reveals features of an early onset mutation in Parkinson’s disease
Source: Commun Biol. 2019 Oct 11;2:374. doi: 10.1038/s42003-019-0598-9 (PMC6789109; doi:10.1038/s42003-019-0598-9)
Supplement: Supplementary file 4 — Reporting Summary [file 42003_2019_598_MOESM4_ESM.pdf]

nature research

Corresponding author(s): Guilherme A. P. de Oliveira

Last updated by author(s): Aug 26, 2019

Reporting Summary

Nature Research wishes to improve the reproducibility of the work that we publish. This form provides structure for consistency and transparency in reporting. For further information on Nature Research policies, see Authors & Referees and the Editorial Policy Checklist.

Statistics

For all statistical analyses, confirm that the following items are present in the figure legend, table legend, main text, or Methods section.

n/a	Confirmed	
☐	☒	The exact sample size (*n*) for each experimental group/condition, given as a discrete number and unit of measurement
☐	☒	A statement on whether measurements were taken from distinct samples or whether the same sample was measured repeatedly
☒	☐	The statistical test(s) used AND whether they are one- or two-sided<br>*Only common tests should be described solely by name; describe more complex techniques in the Methods section.*
☒	☐	A description of all covariates tested
☒	☐	A description of any assumptions or corrections, such as tests of normality and adjustment for multiple comparisons
☐	☒	A full description of the statistical parameters including central tendency (e.g. means) or other basic estimates (e.g. regression coefficient) AND variation (e.g. standard deviation) or associated estimates of uncertainty (e.g. confidence intervals)
☒	☐	For null hypothesis testing, the test statistic (e.g. *F*, *t*, *r*) with confidence intervals, effect sizes, degrees of freedom and *P* value noted<br>*Give P values as exact values whenever suitable.*
☒	☐	For Bayesian analysis, information on the choice of priors and Markov chain Monte Carlo settings
☒	☐	For hierarchical and complex designs, identification of the appropriate level for tests and full reporting of outcomes
☒	☐	Estimates of effect sizes (e.g. Cohen's *d*, Pearson's *r*), indicating how they were calculated

Our web collection on statistics for biologists contains articles on many of the points above.

Software and code

Policy information about availability of computer code

Data collection	Fig1a-d, Fig6c, Fig7a-d, and Fig8b: SoftMax Pro 7.0; Fig1e, Fig4a,b, Fig5b,d, Fig6e, and Fig8b: TEM Imaging and Analysis v5.6; Fig2b: Jasco Spectra Manager; Fig3a: EPU software FEI; Fig5e: ThermoFisher Proteome Discoverer 1.4.1; Fig8c,d: PrimeView v.5.31
Data analysis	Fig1a-d, Fig2b, Fig4c,d, Fig6c, Fig7a-d, and Fig8b,c,d: OriginPro v8.1; Fig1f-m, Fig6a,b,d, Fig7e-h: GraphPad Prism6 v6.01; Fig3b-c: EMAN2 software; Fig5e,f: Software Scaffold 4.8.1 and Keynote v6.6.2;

For manuscripts utilizing custom algorithms or software that are central to the research but not yet described in published literature, software must be made available to editors/reviewers. We strongly encourage code deposition in a community repository (e.g. GitHub). See the Nature Research guidelines for submitting code & software for further information.

Data

Policy information about availability of data

All manuscripts must include a data availability statement. This statement should provide the following information, where applicable:

- Accession codes, unique identifiers, or web links for publicly available datasets
- A list of figures that have associated raw data
- A description of any restrictions on data availability

Source data file is provided and described within the Data availability statement

Field-specific reporting

Please select the one below that is the best fit for your research. If you are not sure, read the appropriate sections before making your selection.

☒ Life sciences ☐ Behavioural & social sciences ☐ Ecological, evolutionary & environmental sciences

Life sciences study design

All studies must disclose on these points even when the disclosure is negative.

Sample size	Sample size was predetermined to a minimum of 5 independent kinetic experiments using at least two protein batches. Additional kinetic traces (at least from two different days) were then performed to read soluble and fibrillar fractions by the end of reactions. N > 9 is provided for each studied condition. For cryo-EM and negative staining, fibrils from two independent batch preparations were imaged. Size exclusion chromatography and circular dichroism was performed twice.
Data exclusions	Outliers from raw data in Suppl Fig. 4 and from all other kinetic traces were excluded during averaging five independent traces. Point exclusion was performed using an automated cutoff procedure in Wolfram Mathematica with no impact to the kinetic tendency behavior. This procedure was done due to so many increment points used during data acquisition (each 3 to 4 min during ~ 5,400 min) as an strategy to capture finer growth tendency.
Replication	All attempts to replicate the data were successful. Some measurements (e.g., thioflavin T measurements at the end of kinetic traces) presented higher variance among replicates, but they are all depicted as dot plots.
Randomization	This is not relevant to this study
Blinding	Blinding to group allocation is not possible in this study because we are evaluating / polishing defined variables influencing amyloid formation based on previous observations from other groups.

Reporting for specific materials, systems and methods

We require information from authors about some types of materials, experimental systems and methods used in many studies. Here, indicate whether each material, system or method listed is relevant to your study. If you are not sure if a list item applies to your research, read the appropriate section before selecting a response.

Materials & experimental systems

n/a	Involved in the study	
☒	☐	Antibodies
☒	☐	Eukaryotic cell lines
☒	☐	Palaeontology
☒	☐	Animals and other organisms
☒	☐	Human research participants
☒	☐	Clinical data

Methods

n/a	Involved in the study	
☒	☐	ChIP-seq
☒	☐	Flow cytometry
☒	☐	MRI-based neuroimaging
